# Bone metastases from head and neck malignancies: Prognostic factors and skeletal-related events

**DOI:** 10.1371/journal.pone.0213934

**Published:** 2019-03-20

**Authors:** Salvatore Grisanti, Susanna Bianchi, Laura D. Locati, Luca Triggiani, Stefania Vecchio, Alberto Bonetta, Cristiana Bergamini, Pierfranco Conte, Mario Airoldi, Marco Merlano, Paolo Carlini, Toni Ibrahim, Ciro Rossetto, Salvatore Alfieri, Paolo Pronzato, Sandro Tonoli, Roberto Maroldi, Piero Nicolai, Carlo Resteghini, Stefano M. Magrini, Alfredo Berruti

**Affiliations:** 1 Medical Oncology Unit, Department of Medical and Surgical Specialties, Radiological Sciences, and Public Health, University of Brescia, ASST Spedali Civili, Brescia, Italy; 2 Medical Oncology/Head and Neck Unit, Fondazione IRCCS Istituto Nazionale dei Tumori, Milan, Italy; 3 Radiation Oncology Unit, Department of Medical and Surgical Specialties, Radiological Sciences, and Public Health, University of Brescia, ASST Spedali Civili, Brescia, Italy; 4 Medical Oncology Unit, IRCCS San Martino, IST National Cancer Institute and University of Genoa, Genoa Italy; 5 Department of Radiotherapy, Istituti Ospitalieri di Cremona (ASST), Cremona, Italy; 6 Department of Surgery, Oncology and Gastroenterology, University of Padua, Division of Medical Oncology 2, Istituto Oncologico Veneto IRCCS, Padua, Italy; 7 2nd Medical Oncology Division, Città della Salute e della Scienza Hospital of Turin, Italy; 8 Medical Oncology, A.O. S. Croce and Carle Teaching Hospital, Cuneo, Italy; 9 Medical Oncology, Istituto Nazionale Tumori Regina Elena, Rome, Italy; 10 Osteoncology and Rare Tumors Center, Istituto Scientifico Romagnolo per lo Studio e la Cura dei Tumori (IRST) IRCCS, Meldola, Italy; 11 Department of Oncology, University Hospital Santa Maria della Misericordia, Udine, Italy; 12 Radiology Unit, Department of Medical and Surgical Specialties, Radiological Sciences, and Public Health, University of Brescia, ASST Spedali Civili, Brescia, Italy; 13 Unit of Otorhinolaryngology–Head & Neck Surgery, Department of Medical and Surgical Specialties, Radiological Sciences, and Public Health, ASST Spedali Civili, Brescia, Italy; George Washington University, UNITED STATES

## Abstract

**Background:**

We conducted a multicenter retrospective analysis to describe the characteristics, frequency of skeletal-related events (SREs), and prognosis of head and neck cancer (HNC) in patients with bone metastases (BM).

**Patients and methods:**

The data of 192 HNC patients with BMs were collected. Analyses were conducted separately in 64 nasopharyngeal cancer (NPC) patients and in 128 non-NPC patients.

**Results:**

SREs occurred in 34 (27%) non-NPC and in 6 (9%) NPC patients, respectively. Median overall survival (OS) was 25 and 6 months in NPC and non-NPC patients, respectively. Locoregional recurrence (hazard ratio [HR] 2.33, 95% confidence interval (CI) 1.1–4.93), synchronous BM (HR 0.25, 95% CI 0.59–0.71) and bone-directed therapies (BDT) (HR 0.26, 95% CI 0.10–0.68) were independent prognostic factors for OS in NPC patients. Combined bone radiotherapy (RT) and BDT in NPC patients obtained longer survival (38 months) than either therapy alone (25 months) or neither of these therapies (8 months).

**Conclusions:**

Patients with BMs from non-NPC have a poor prognosis and are at high risk of SREs. NPC patients with BMs are at relatively low risk of SREs. BDT may potentially improve survival, particularly when combined with bone RT. This last finding deserves prospective confirmation.

## Introduction

Epithelial neoplasms arising in the head and neck are the sixth most frequent cancer worldwide, with an incidence of 560,000 cases/year, albeit with large geographical heterogeneity [1]. While concomitant cisplatin-based chemoradiotherapy (CRT) has improved survival and organ preservation in patients with locally advanced disease [2], disease recurs either locoregionally or at distant sites in more than 50% of cases.

The lungs are the most common distant metastatic site, followed by extracervical lymph nodes, bone, liver, and skin [3]. While bone metastases have been generally considered a rare and late event in patients with head and neck cancer (HNC), the reported incidence varies with tumor site, ranging from 50%-80% in patients with nasopharyngeal carcinoma (NPC) [4,5] to 2%-22% in squamous cell non-NPC [3,6–8]. The prognosis of HNC patients with bone metastases is reportedly poor and the expected overall survival often does not exceed 8 months [7,9]. Furthermore, bone involvement greatly diminishes quality of life since it causes bone pain and skeletal-related events (SREs) such as bone fractures, spinal cord compression, and hypercalcemia [10].

The management of patients with bone metastases requires a multidisciplinary team involving medical oncologists and radiation oncologists, as well as orthopedic surgeons, neurosurgeons, and interventional radiologists to provide the best treatment strategy, appropriate preventive measures, and treatment of SREs. Bone-directed therapies (BDT), zoledronic acid (ZA) and denosumab (D), have been demonstrated to significantly reduce the risk of SREs in patients with bone metastases from prostate, breast, and lung cancer, as well as other solid neoplasms and multiple myeloma [11]. In a large retrospective study involving patients with bone metastases from NPC, the addition of ZA to chemotherapy was found to be associated with a lower proportion of SREs and improved survival in comparison to chemotherapy alone [12].

To date, the prognostic factors and management of bone metastases in patients with HNC have never been studied in detail due to their rarity. Therefore, this retrospective multicenter study was undertaken to describe the natural history and the pattern of care of HNC patients with bone metastases. The secondary aims were to identify prognostic factors and to explore the impact of antineoplastic and BDT on patient survival.

## Patients and methods

### Study design

This retrospective, observational study was conducted at 11 cancer centers in Italy. Briefly, patients were considered eligible for enrollment if: they had a known diagnosis of HNC (all histological types were allowed), including nasopharyngeal carcinoma (NPC) and paranasal sinus cancer; at least one distant bone metastasis not contiguous with the primary tumor was present (i.e., a lesion for which infiltration by direct contiguity with the primary tumor could be excluded);—imaging records and follow-up data were available. Exclusion criteria were: a diagnosis of either carcinoma of the thyroid gland and/or of the salivary glands; and the presence of a synchronous neoplasm different from HNC. Bone metastases were identified on the basis of surgical records, bone biopsies or imaging techniques including X-ray, ^99^Tc-bone scintigraphy, magnetic resonance imaging (MRI), computed tomography (CT), and whole body ^18^fluoro-deoxy-glucose positron-emission tomography (18F-FDG-PET/CT). The AJCC staging system 7^th^ edition was retrospectively applied for staging classification. Clinical variables included age, sex, site of the primary tumor in the head and neck, histology, primary treatment, limited vs. extensive bone involvement (1 site vs. ≥2 sites), locoregional recurrence, and visceral metastases. Skeletal-related events (SREs) were bone fractures, spinal cord and nerve root compression, and hypercalcemia.

The study was approved by the Institutional Review Board of the Coordinating Center in Brescia on 3 December 2014 (Comitato Etico Provinciale Provincia di Brescia c/o Spedali Civili [Study n. NP1848 –Studio SURMOS]) and of each participating institution, here enlisted: Comitato per la Sperimentazione dei Nuovi Metodi Diagnostici e Terapeutici c/o IRCCS Istituto Nazionale Tumori di Milano, Comitato Etico IRCCS AOU San Martino-IST, Comitato Etico Val Padana c/o ASST di Cremona, Comitato Etico per la Sperimentazione Clinica (CESC) c/o Istituto Oncologico Veneto IRCCS, Comitato Etico Interaziendale A.O.U. Città della Salute e della Scienza di Torino—A.O. Ordine Mauriziano—A.S.L. Città di Torino c/o Città della Salute e della Scienza (c/o Presidio Ospedaliero Molinette), Comitato Etico Interaziendale dell’AO S. Croce e Carle di Cuneo, Comitato Etico Sezione I.F.O. (Istituti Regina Elena e San Gallicano) di Roma, Comitato Etico della Romagna (CEROM) c/o Istituto Scientifico Romagnolo per lo Studio e la Cura dei Tumori (IRST) IRCCS, Comitato Etico Unico Regionale (CEUR) c/o Azienda Regionale di Coordinamento per la Salute (ARCS) della Regione Friuli Venezia Giulia.

The study was conducted in accordance with the Declaration of Helsinki for clinical studies.

### Statistical analysis

Given the retrospective, observational nature of the study, no pre-specified hypothesis was formulated and no formal sample size was calculated. NPC patients were analyzed separately from the other HNC patients based on the different tumor histology and clinical behavior.

Outcome measures were: time-to-first bone metastases calculated from the initial diagnosis of HNC to the onset of bone metastases; time-to-SRE calculated from the diagnosis of bone metastasis to the occurrence of bone fracture or spinal cord compression or hypercalcemia. Patients in which no SRE was recorded were considered censored at death or last follow-up; bone metastases-specific survival (BM-OS) was calculated from the diagnosis of bone metastasis to death or last follow-up. The Kaplan-Meier method was used to generate survival curves and the Mantel-Haenzel log-rank test to establish differences among groups. Analysis of the distribution of clinical variables was descriptive. Categorical clinical variables were analyzed with the chi-square test or Fisher’s exact test when appropriate. Cut-off points were identified for continuous variables based on the median value. The potential impact of single clinical variables on outcome measures was estimated by calculating the hazard ratio (HR) and its 95% confidence interval (CI) in a univariate Cox analysis. Variables significantly associated with outcome measures (time to SRE or BM-OS) at a p level of significance ≤.1 were included in a multivariate Cox model. Heterogeneity in the effect of BDT in patient subgroups was evaluated using standard tests for interaction. Statistical analysis was performed using SPSS (IBM SPSS Statistics for Windows, Version 22.0. Armonk, NY: IBM Corp.). All p values are two-sided. P values less than .05 were considered statistically significant.

## Results

### Patient characteristics

The data from 192 HNC patients with bone metastases (64 NPCs and 128 non-NPCs) were collected from 11 cancer centers in Italy between 2008 and 2016. Table 1 presents patient characteristic.

**Table 1:** Patient characteristics.

Characteristic	NPC patients (N = 64) No. (%)	Non-NPC patients (N = 128) No. (%)
**Median age (range), years**	49 (15–71)	61 (28–87)
<50 years	33 (52)	17 (13)
≥50 years	31 (48)	111 (87)
**Sex**		
Male	46 (72)	102 (80)
Female		18 (28)	26 (20)
**Race**		
Caucasian	59 (92)	126 (98)
Asian	3 (5)	1 (1)
Hispanic	0 (0)	1 (1)
Black	2 (3)	0 (0)
**HNC anatomical district**		
nasopharynx	64 (100)	-
oropharynx/oral cavity	-	61 (48)
larynx	-	25 (19)
hypopharynx	-	13 (10)
paranasal sinuses	-	6 (5)
nasal sinus/ethmoid	-	10 (8)
other	-	13 (10)
**Histology**		
squamous	4 (6)	107 (84)
undifferentiated	55 (86)	8 (6)
adenocarcinoma	5 (8)	4 (3)
other	-	9 (7)
**Tumor grade**		
G1	-	6 (5)
G2	6 (6)	29 (23)
G3	11 (17)	67 (52)
G4	45 (71)	10 (8)
NA	4 (6)	16 (12)
**AJCC stage at diagnosis**		
limited (I-II)	3 (5)	17 (13)
locally-advanced (III-IVa-b)	36 (56)	91 (71)
metastatic (IVc)	25 (39)	20 (16)
**Metastatic pattern**		
locoregional LN	32 (50)	79 (62)
visceral	29 (45)	79 (62)
bone	64 (100)	128 (100)
**No. of metastatic sites**		
1	21 (33)	34 (27)
2	20 (31)	46 (36)
>3	23 (36)	48 (37)
**Skeletal involvement**		
limited (1 site)	27 (42)	69 (54)
extensive (≥2 sites)	37 (58)	59 (46)
**Timing of bone metastases**		
synchronous	10 (16)	10 (8)
metachronous	54 (84)	118 (92)
**Surgery** (open surgery, vertebroplasty, radiofrequency)	2 (3)	5 (4)
**Bone radiotherapy**	45 (70)	68 (53)
**Chemotherapy**	51 (80)	92 (72)
**Bone-directed therapies**	23 (35)	42 (33)
Biphoshonates	16 (25)	37 (29)
Denosumab	3 (4)	1 (1)
Both	4 (6)	4 (3)
**Other treatments (orthopedic devices)**	14 (22)	23 (18)

NA = not available; LN = lymph nodes; NPC = nasopharyngeal carcinoma.

The median age was 49 years (range 15–71) and 61 years (range 28–87) in the NPC and the non-NPC patients, respectively. Among the 64 NPC patients, 55 (86%) had an undifferentiated carcinoma. Among the 128 non-NPC patients, 107 (84%) had squamous cell carcinoma and 21 (16%) had other histologies (6% undifferentiated, 3% adenocarcinoma, 7% other); the most frequent anatomical site of the primary tumor was the oropharynx/oral cavity, followed by the larynx, hypopharynx, and sinonasal cavities. Stratification by staging at first diagnosis revealed locally advanced disease (American Joint Committee on Cancer [AJCC] stage III, IVa, IVb) in 56% and 71% of NPC and non-NPC patients, respectively.

At diagnosis, 25 (39%) NPC and 20 (16%) non-NPC patients presented with metastatic disease: bone metastases were present in 10 patients in each group (16% and 8%, respectively); visceral metastases in 29 (45%) NPC and in 79 (62%) non-NPC patients; and locoregional recurrence in 32 (50%) NPC and in 79 (62%) non-NPC patients. In patients with no bone metastases at diagnosis, the median time-to-first bone metastases was 9 months (range 6.1–11.8) in the NPC and 12 months (range 8.7–15.2) in the non-NPC patients. In both groups, this interval was significantly influenced by the initial tumor stage. When we stratified patients as having limited, locally advanced or metastatic disease, the median time was 25, 13, or <1 month (p < .0005) in the NPC and 38, 12, and 2 months in non-the NPC patients, respectively (p < .0005)

Overall, the burden of bone involvement was limited (1 site) in 27 (42%) and extensive (≥2 sites) in 37 (58%) NPC patients; it was limited to 1 site in 69 (54%) non-NPC patients and extensive in 59 (46%). Bone metastases were mainly localized to the spine (61%), pelvic bones (15%), ribs (8%) or other sites (16%), without significant differences between the NPC and the non-NPC patients.

### Skeletal-related events and pattern of care

**Fig 1:**
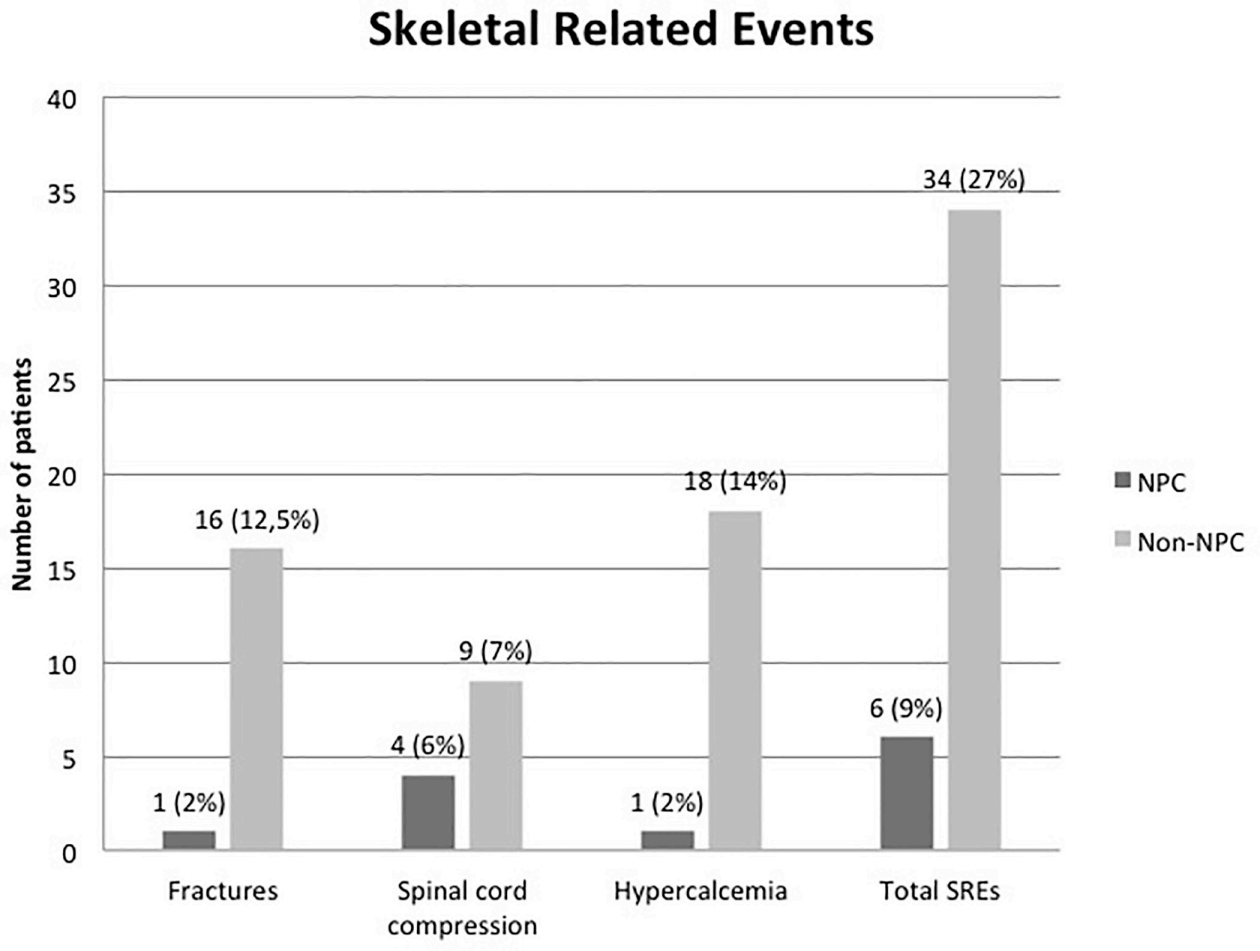
SRE frequency and distribution in NPC and non-NPC patients.

SREs occurred in 34 (27%) non-NPC patients and in 6 (9%) NPC patients, respectively (Fig 1). Hypercalcemia was the most frequently observed SRE in the non-NPC patients (14%), followed by bone fractures (12%) and spinal cord compression (7%). Spinal cord compression was the most frequent SRE in the NPC patients (6%).

Following the diagnosis of bone metastases, the majority (92%) of patients received early multimodal treatment with a combination of chemotherapy (NPC 80%, non-NPC 72%), radiotherapy (NPC 70%, non-NPC 53%) and BDT, with ZA or D (NPC 35%, non-NPC 33%). No serious BDT-related complications were observed. Surgical treatment of bone metastases (laminectomy, radiofrequency ablation of bone lesions, vertebroplasty) was performed only in cases of bone fracture or spinal cord compression (NPC 3%, non-NPC 4%). Other treatments included the use of orthopedic devices (busts, braces, and crutches) in 22% of the NPC and 18% of the non-NPC patients (Table 1).

### Survival outcomes and analysis of prognostic factors

**Fig 2:**
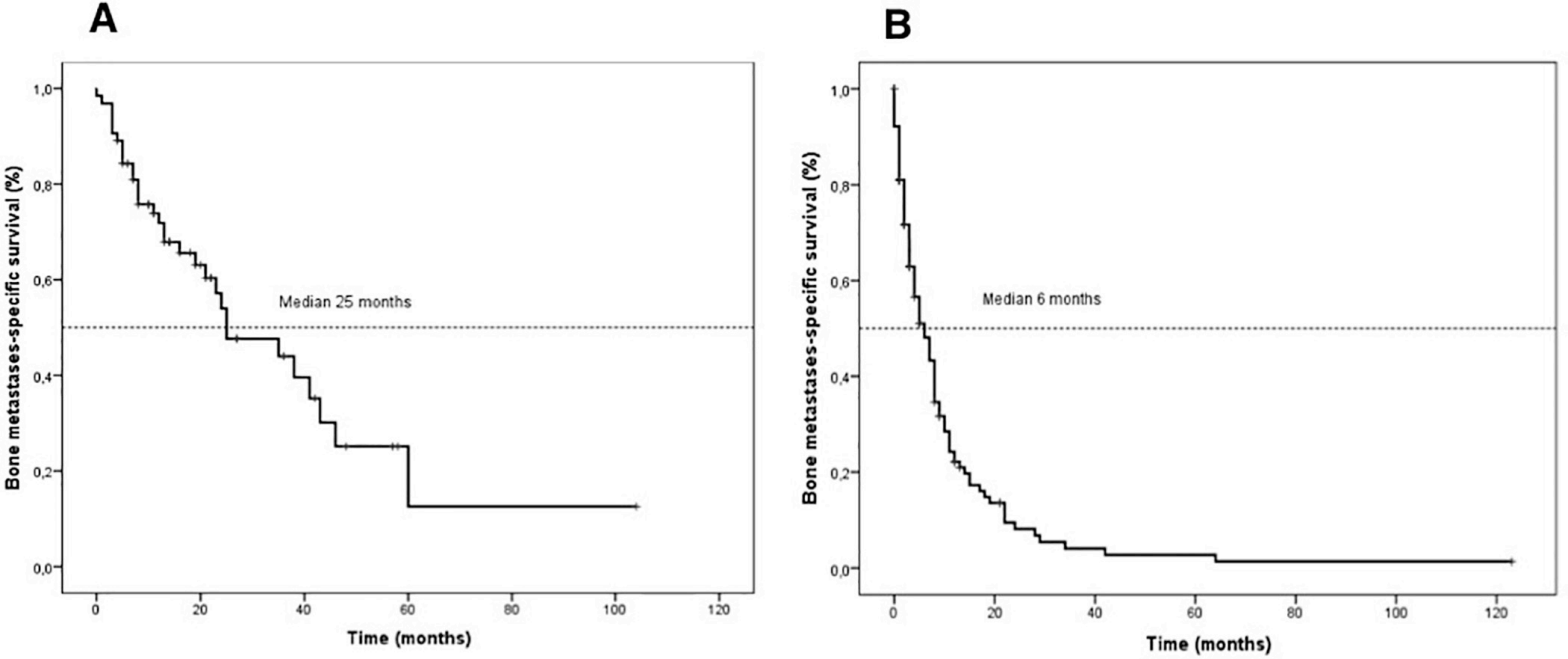
Kaplan-Meier curves of bone metastases-specific survival in the NPC (A) and the non-NPC (B) patients.

After a median follow-up of 47 months, 32 (50%) NPC and 103 (80%) non-NPC patients had died. The median BM-OS was 25 (95% CI 11.06–38.94) and 6 (95% CI 3.92–8.07) months for the NPC and the non-NPC patients, respectively (Fig 2A and 2B). Further skeletal progression after diagnosis of bone metastasis was observed in 55% of patients with NPC and in 40% of patients with non-NPC. In 80% and 70% of the respective groups skeletal progression was part of systemic disease progression. The median progression-free survival (PFS) was 11 months in the NPC patients and 5 months in the non-NPC patients.

We then analyzed the clinical and pathological characteristics as determinants of BM-OS in both groups. In the NPC patients, the presence of synchronous metastases (HR 0.31, 95% CI 0.094–1.043, p .059), bone RT (HR 0.318, 95% CI 0.149–0.678, p .003), and BDT (HR 0.348, 95% CI 0.142–0.852, p .021) was significantly associated with a lower risk of death at univariate analysis. Locoregional recurrence (HR 1.86, 95% CI 0.906–3.827, p .091) just failed to be statistically correlated, while the other variables (age, sex, tumor stage, histology, number of metastatic sites, visceral metastases, SREs, surgery, chemotherapy, and other treatments) showed no relationship with survival. At multivariate analysis, locoregional recurrence, synchronous bone metastases, and BDT were independent prognostic factors for survival (Table 2).

**Table 2:** Uni-/Multivariate analyses of prognostic factors for bone metastases-specific survival in NPC patients.

Variable	Univariate analysis				Multivariate analysis		
	Deaths/n	HR	95% CI	p	HR	95% CI	p
**Age**							
<50	13/33	1.00	.785–3.229	.197	**-**	**-**	**-**
≥19	19/31	1.59			**-**		
**Sex**							
Male	21/46	1.00	.856–1.797	.256	**-**	**-**	**-**
Female	11/18	1.24			**-**		
**Stage at diagnosis**							
Lim	2/3	1.00			-	-	-
LA	19/36	0.36	.081–1.620	.184	-		
M+	11/25	0.39	.086–1.841	.239	-		
**Histology**							
undifferentiated	27/55	1.00	.177–6.522	.937	-	-	-
other	5/9	1.07			-		
**No. of metastatic sites**							
1	10/21	1.00				-	-
2	9/20	1.62	.318–1.948	.605	-		
>2	23/23	0.79	.699–3.768	.260	-		
**Bone involvement**							
limited	13/27	1.00	.624–2.623	.502	-	-	-
extensive	19/37	1.28			-		
**Visceral mets**							
no	17/35	1.00	.751–3.097	.243	-	-	-
yes	15/29	1.52			-		
**Locoregional LN mets**							
no	12/32	1.00	.906–3.827	.091	1.00	1.10–4.933	.027
yes	20/32	1.86			2.33		
**Bone mets**							
metachronous	29/54	1.00	.094–1.043	.059	1.00	.059-.715	.013
synchronous	3/10	0.31			.255		
**All SREs**							
no	29/50	1.00	380–4.238	.698	-	-	-
yes	3/6	1.26			-		
**Fractures**							
no	31/63	1.00	.511–29.606	.190	-	-	-
yes	1/1	3.89			-		
**Spinal cord compression**							
no	31/60	1.00	.067–3.645	.489	-	-	-
yes	1/4	.49			-		
**Hypercalcemia**							
no	31/63	1.00	.669–40.193	.115	-	-	-
yes	1/1	5.18			-		
**Surgery for bone mets**							
no	31/62	1.00	.189–10.498	.739	-	-	-
yes	1/2	1.407			-		
**RT for bone mets**							
no	12/19	1.00	.149-.678	.003	1.00	.242–1.153	.109
yes	20/45	.318			.529		
**CT for bone mets**							
no	8/13	1.00	.338–1.702	.502	-	-	-
yes	24/51	.758			-		
**Bone-directed therapies**							
no	26/41	1.00	.142-.852	.021	1.00	.105-.679	.006
yes	6/23	.348			.267		

M+: metastatic; LA: locally advanced; Lim: limited; Mets: metastases; SRE: skeletal-related event; RT: radiotherapy; CT: chemotherapy; NPC: nasopharyngeal carcinoma; HR: hazard ratio.

In the non-NPC patients, the presence of locoregional recurrence (HR 1.84, 95% CI 1.215–2.811, p .004) and bone RT (HR .676, 95% CI 0.453–1.007, p .054) significantly influenced overall survival at univariate analysis, whereas the other covariates did not. At multivariate analysis, locoregional recurrence was the only independent variable associated with patient prognosis (Table 3).

**Table 3:** Uni-/Multivariate analyses of prognostic factors for bone metastases-specific survival in non-NPC patients.

Variable	Univariate analysis				Multivariate analysis		
	Deaths/n	HR	95% CI	p	HR	95% CI	p
**Age**							
<50	15/17	1.00	.430–1.304	.307	-	-	-
≥50	88/111	0.74			-		
**Sex**							
Male	81/102	1.00	.934–1.500	.163	-	-	-
Female	22/26	1.18			-		
**Stage at diagnosis**							
Lim	15/17	1.00			-	-	-
LA	73/91	0.88	.506–1.543	.735	-		
M+	15/20	1.13	.551–2.326	.663	-		
**Histology**							
squamous	88/107	1.00	.605–1.818	.864	-	-	-
other	15/21	1.04			-		
**HNC anatomical district** (oropharynx/oral cavity vs. larynx vs. hypopharynx vs. paranasal sinuses vs. nasal sinus/ethmoid vs. other)		ns	ns	ns	-	-	-
**No. of metastatic sites**							
1	24/34	1.00			-		
2	38/46	1.02	.611–1.714	.930	-	-	-
>2	41/48	1.49	.903–2.489	.118	-	-	-
**Bone involvement**							
limited	55/69	1.00	.728–1.583	.722	-	-	-
extensive	48/59	1.07			-		
**Visceral mets**							
no	37/49	1.00			-	-	-
yes	66/79	1.13	.757–1.704	.540	-		
**Locoregional LN mets**							
no	35/49	1.00	1.215–2.811	.004	1.00	1.126–2.671	.012
yes	68/79	1.84			1.73		
**Bone mets**							
metachronous	95/118	1.00	.460–2.145	.986	-	-	-
synchronous	7/10	1.00			-		
**All SREs**							
no	72/94	1.00	.892–2.095	.151	-	-	-
yes	31/34	1.36			-		
**Fractures**							
no	88/112	1.00	.582–1.757	.969	-	-	-
yes	15/16	1.01			-		
**Spinal cord compression**							
no	94/119	1.00	.658–2.609	.441	-	-	-
yes	9/9	1.31			-		
**Hypercalcemia**							
no	87/110	1.00	.800–2.343	.251	-	-	-
yes	16/18	1.37			-		
**Surgery for bone mets**							
no	98/123	1.00	.335–2.040	.679	-	-	-
yes	5/5	.827			-		
**RT for bone mets**							
no	50/60	1.00	.453–1.007	.054	1.00	.512–1.161	.213
yes	53/68	.676			.771		
**CT for bone mets**							
no	26/36	1.00	.542–1.345	.496	-	-	-
yes	77/92	.854			-		
**Bone-directed therapies**							
no	65/86	1.00	.663–1.487	.973	-	-	-
yes	38/42	.993			-		

M+: metastatic; LA: locally advanced; Lim: limited; Mets: metastases; SRE: skeletal-related event; RT: radiotherapy; CT: chemotherapy; NPC: nasopharyngeal carcinoma; HR: hazard ratio.

### Predictive factors of skeletal-related events

Among the NPC patients, only female gender significantly correlated with a higher risk of SRE at both univariate (HR 7.0, 95% CI 1.28–37.32, p .025) and multivariate (HR 5.95, 95% CI 1.04–34.04, p .045) analysis (S1 Table). Among the non-NPC patients, locoregional recurrence (HR 2.13, 95% CI 0.992–4.603, p .052) and chemotherapy (HR 2.22, 95% CI 1.102–4.479, p .026) were directly associated with an increased risk of SRE at univariate analysis, whereas BDT (HR 0.244, 95% CI 0.121–0.494, p < .0005) showed an inverse relationship. However, only locoregional recurrence maintained a significant independent role at multivariate analysis (S2 Table).

### Effect of combined radiotherapy and bone-directed therapies on survival

Given the significant impact of RT and BDT on prognosis for the NPC patients at univariate analysis, we investigated an interaction effect between these two treatment modalities. At bi-variate analyses, BDT were associated with a similar improvement in survival for both RT-treated and non-RT-treated patients with bone metastases (S1 Fig); therefore, the test of interaction was negative (p = 0.62).

**Fig 3:**
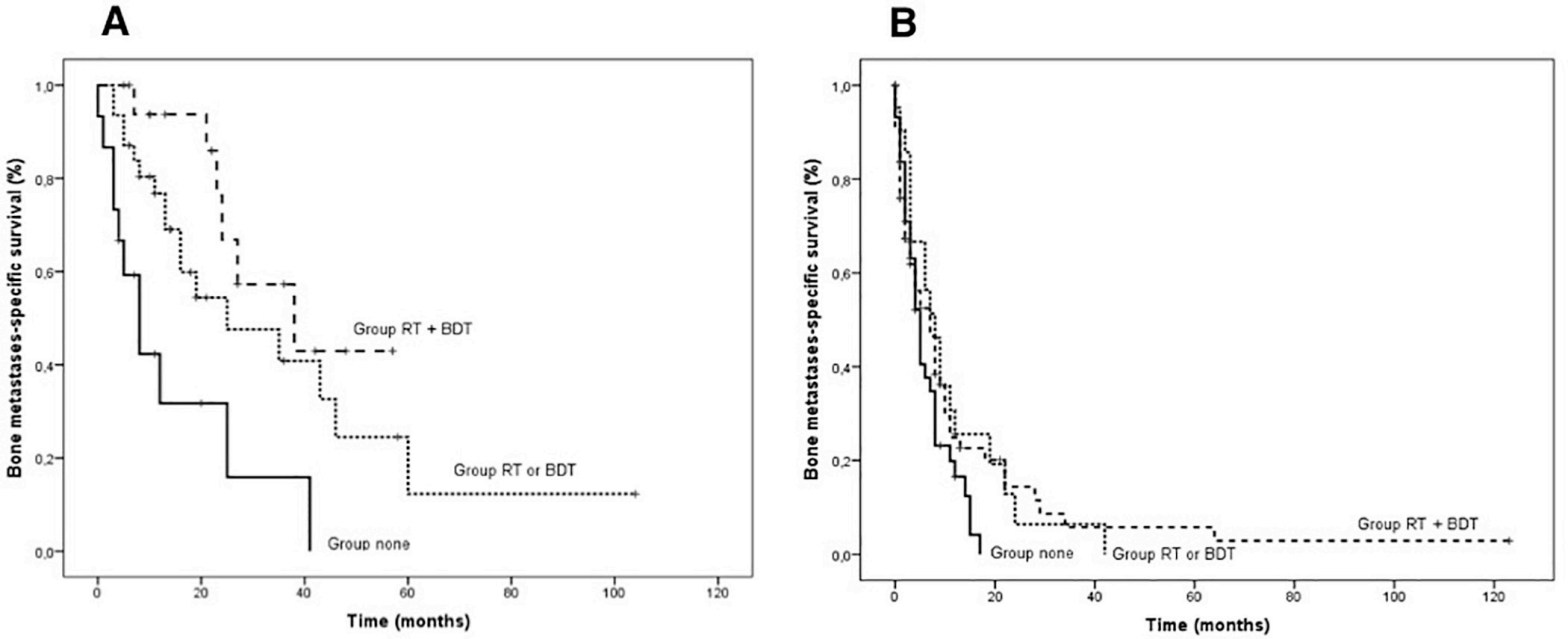
Kaplan-Meier curves of bone metastases-specific survival stratified by treatment in NPC (A) and non-NPC (B) patients.

We then generated Kaplan-Meier curves in which patients were stratified in three groups according to whether they received both RT and BDT (group Both) or only one of the two therapeutic modalities (group Only one) or neither form of therapy (group None). Among the NPC patients, there was a statistically significant difference between the three groups in favor of the combined therapy group (median BM-OS 38 vs. 25 vs. 8 months, p .007 and .001) (Fig 3A). Statistical significance was maintained after adjusting for locoregional recurrence. We also explored the role of RT alone after adjusting for the burden of bone involvement. Among the NPC patients with limited but not extensive bone involvement, RT was associated with a statistically significant reduction in the risk of death (median BM-OS 48 vs. 8 months, HR .174, 95% CI 0.057–0.530, p .002) without a significant interaction effect (p .163) between covariates (S2 Fig).

Among the non-NPC patients, the Kaplan-Meier curves displayed a statistically non-significant difference between the three groups in favor of the combined therapy group (median BM-OS 8 vs. 7 vs. 5 months, p .852 and .056) (Fig 3B).

## Discussion

Head and neck cancer comprises a variety of tumors that differ in biology and clinical management. In this heterogeneous group of cancers, bone has been reported to be the first and second most frequent metastatic site in NPC and non-NPC patients, respectively [3]. Bone metastasis is considered a rare and late event in patients with non-NPC. Few studies to date have investigated bone metastases in detail [6–8]: from the more than 9,300 patients included in these studies, only 36 (0.38%) patients with bone metastases were recorded, too small a number to obtain significant indications.

Here, we analyzed a series of HNC patients selected on the basis of overt bone metastases to describe their clinical characteristics and the pattern of care outside a clinical trial. Approximately 16% of NPC patients and 8% of non-NPC patients had bone metastatic disease at diagnosis. Based on this observation, however, full skeletal imaging cannot be recommended for initial staging of disease in NPC and non-NPC patients.

For the other patients without metastases at presentation, the median time-to-first bone metastasis was 9 months among the NPC and 12 months among the non-NPC patients. Such a short interval indicates that in HNC patients likely to develop bone metastases, this event occurs early in the natural history of the disease. This observation is not surprising since we have noted that circulating tumor cells (CTCs), which are a putative expression of cancer cells eventually located in bone in a dormant state [13,14], are detected in 15% of non metastatic HNC patients [15]. Indeed, the presence of CTC in HNC patients portends an overall poorer prognosis [16].

In the NPC and the non-NPC patients, bone metastases were not isolated but rather were part of systemic progression of disease that included locoregional recurrence and visceral metastases. As a consequence, systemic chemotherapy was started early after diagnosis of metastatic bone disease. In many cases, BDT (bisphosphonates and denosumab) were introduced after the first SRE. Bone surgery was performed when spinal cord compression or bone fractures developed.

In prospective, randomized clinical trials testing the efficacy of bone resorption inhibitors, the definition of SRE, besides skeletal complications, includes also therapeutic modalities for the management of SREs, such as orthopedic/neurosurgical intervention and RT. In the present study, however, we used a stricter definition of SREs that enabled us to distinguish true skeletal complications from their treatment. Based on these criteria, we found a low number of true SREs among the NPC patients (9%), whereas the rate of SREs among the non-NPC (27%) patients was similar to that observed in other multicenter retrospective series involving patients with bone metastasis from lung (26.4%) [17] and kidney cancer (24%) [18] but lower than that observed in thyroid cancer patients [19] who have a relatively long survival period predisposing them to SREs. As expected, hypercalcemia was more common among the non-NPC patients (14%) due to the frequent secretion of parathyroid related peptides (PTHrP) by tumors with squamous cell histology [20]. Among the NPC patients, female gender was the strongest predictor for shorter time-to-SRE. This result must be interpreted with caution due to the very low number of events; however, experimental and clinical evidence has indicated that female sex hormones may contribute to inducing bone-specific metastases [21–23].

Among the non-NPC patients, only the absence of chemotherapy was associated with a shorter time-to-SRE, while female gender and a higher number of metastatic lesions just failed to attain statistical significance. The administration of BDTs after the first occurrence of an SRE accounts for the association with a shorter time-to-SRE.

The median bone metastasis-specific survival in NPC patients (25 months) was shorter than that reported in another series (33 months) [24], while the median survival of 6 months in the non-NPC patients was similar to that reported in a series of squamous cell carcinoma of HN with bone metastases [6–8]. The occurrence of SREs in our series did not influence BM-OS in either the NPC or the non-NPC patients. However, the presence of locoregional recurrence and the overall treatment for bone metastases predicted clinical outcome at both uni- and multivariate analyses. We found that RT to the bone and the use of BDT (ZA and D) contributed more than other treatment modalities to influencing BM-OS. The effect of RT was statistically significant at univariate but not at multivariate analysis for NPC patients and non-NPC patients alike. BDT were independently associated with better survival in the NPC but not in the non-NPC patients. A possible explanation for the selective advantage of BDT in the NPC patients comes from the observation that the receptor activator of NF-kB (RANK), a key mediator of bone metastases, is highly expressed at the tissue level in 100% of metastatic lesions from NPC [25].

Noteworthy, in NPC patients, although no interaction effect was observed between RT and BDT treatments, the combination of both RT and BDT identified a subgroup of metastatic NPC patients with better prognosis. Furthermore, RT alone in NPC patients with limited bone involvement exerted a survival advantage (S2 Fig). This observation is striking because it indicates an additive effect of the two bone treatments in overall disease control. Similar results were reported by Cao et al., who described the superiority of chemotherapy plus bone RT over chemotherapy alone in low-risk NPC patients with bone metastases [24]. However, the study gave no information about the use of BDT. Further insights come from recent observations of high local control rates and a long-term survival advantage in several solid neoplasms, including NPC patients treated with stereotactic body radiotherapy (SBRT) for oligometastatic disease [26]. On this basis and considering the high frequency of SREs in the subset of non-NPC patients, our position is to start BDT in all HNC patients at initial diagnosis of bone metastases.

## Conclusions

Here, we describe the clinical presentation and pattern of care of bone metastases in HNC in a large multicenter retrospective analysis. To the best of our knowledge, only single institution series have been published to date and none of them was designed to focus on the natural history of bone metastases. The present study has the limitations of a retrospective study, with missing data on important issues such as performance status, human Papilloma Virus (HPV)-status stratification of oropharyngeal (OPC) carcinomas and biochemical parameters. Nevertheless, a few clear messages emerge: patients with bone metastases from non-NPC have a poor prognosis and are at high risk of SREs (hypercalcemia in particular). By contrast, SREs are relatively rare in NPC patients with bone metastases, despite their longer survival. In this latter setting there is a chance for bone-directed treatments (BDT and RT) to improve survival. However, these data are hypothesis-generating and deserve confirmation in a prospective study.

## Supporting information

S1 Fig Kaplan-Meier curves of bone metastases-specific survival bi-stratified by bone-directed therapies (yes = solid line; no = dashed line) with (A) or without (B) radiotherapy in NPC patients.
(TIF)

S2 Fig Kaplan-Meier curves of bone metastases-specific survival bi-stratified by bone radiotherapy (yes = solid line; no = dashed line) and burden of bone involvement (A: limited bone involvement; B = extensive bone involvement) in NPC patients.
(TIF)

S1 Table Uni-/Multivariate analyses of predictive factors for SREs in NPC patients. M+: metastatic; LA: locally advanced; Lim: limited; Mets: metastases; SRE: skeletal-related event; RT: radiotherapy; CT: chemotherapy;
NPC: nasopharyngeal carcinoma; HR: hazard ratio; NA: not assessable.
(PDF)

S2 Table Uni-/Multivariate analyses of predictive factors for SREs in non-NPC patients. M+: metastatic; LA: locally advanced; Lim: limited; Mets: metastases; SRE: skeletal-related event; RT: radiotherapy; CT: chemotherapy;
NPC: nasopharyngeal carcinoma; HR: hazard ratio.
(PDF)

S1 Dataset Anonymized clinical data set of 192 NPC and non-NPC patients analysed. (XLSX)
