# Supplementary figures and images for: Bone metastases from head and neck malignancies: Prognostic factors and skeletal-related events
Source: PLoS One. 2019 Mar 20;14(3):e0213934. doi: 10.1371/journal.pone.0213934 (PMC6426213; doi:10.1371/journal.pone.0213934)

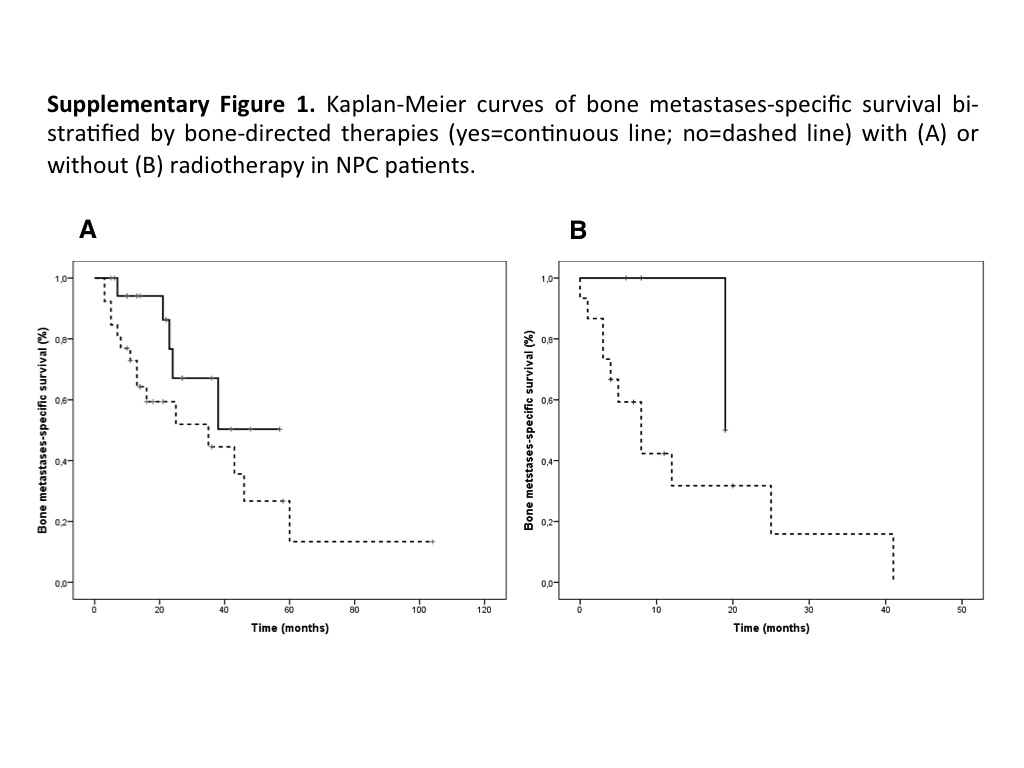

Supplement: S1 Fig — Kaplan-Meier curves of bone metastases-specific survival bi-stratified by bone-directed therapies (yes = solid line; no = dashed line) with (A) or without (B) radiotherapy in NPC patients. (TIF) [file pone.0213934.s001.tif]

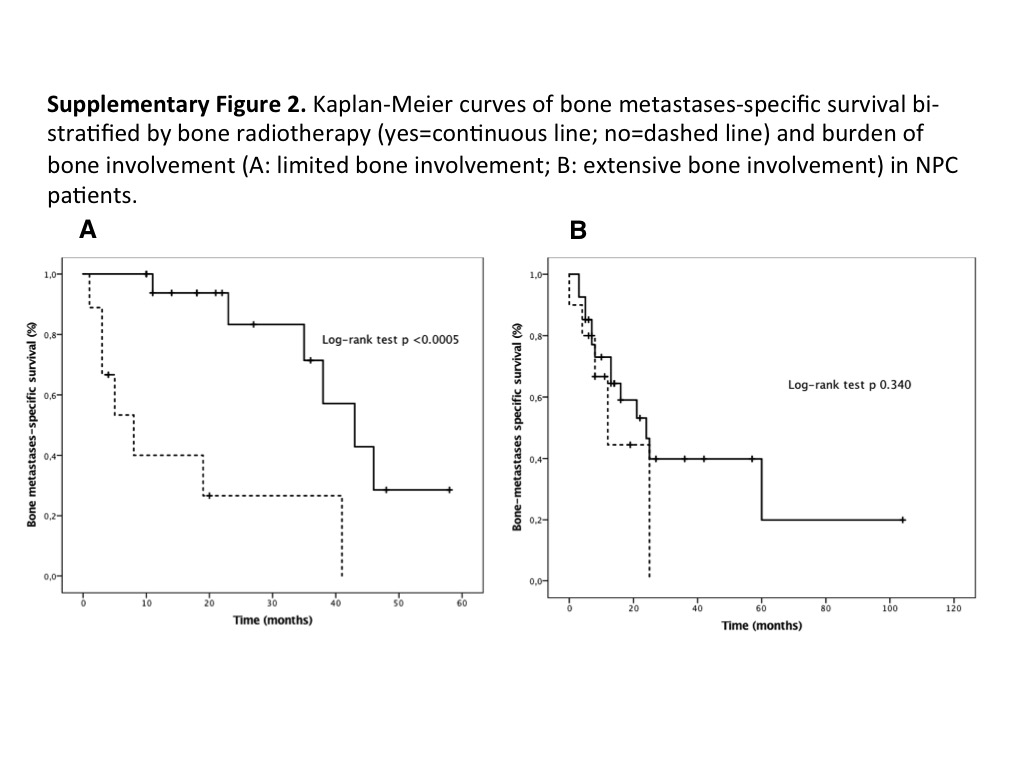

Supplement: S2 Fig — Kaplan-Meier curves of bone metastases-specific survival bi-stratified by bone radiotherapy (yes = solid line; no = dashed line) and burden of bone involvement (A: limited bone involvement; B = extensive bone involvement) in NPC patients. (TIF) [file pone.0213934.s002.tif]
