# Supplementary material for: Bone metastases from head and neck malignancies: Prognostic factors and skeletal-related events
Source: PLoS One. 2019 Mar 20;14(3):e0213934. doi: 10.1371/journal.pone.0213934 (PMC6426213; doi:10.1371/journal.pone.0213934)
Supplement: S1 Table — M+: metastatic; LA: locally advanced; Lim: limited; Mets: metastases; SRE: skeletal-related event; RT: radiotherapy; CT: chemotherapy; NPC: nasopharyngeal carcinoma; HR: hazard ratio; NA: not assessable. (PDF) [file pone.0213934.s003.pdf]

Supplementary Table 1. Uni-/Multivariate analyses of predictive factors for SRE in NPC patients.

Variable	Univariate analysis				Multivariate analysis		
	SRE/n	HR	95% CI	p	HR	95% CI	p
Age <50<br>≥50	4/32<br>2/31	1.00<br>0.45	.082-2.472	.359			
Sex Male<br>Female	2/45<br>4/18	1.00<br>7.0	1.28-37.32	.025	1.00<br>5.95	1.04-34.04	.045
Stage at diagnosis Lim<br>LA<br>M+	1/3<br>3/36<br>2/24	1.00<br>0.11<br>0.14	 0.010-1.239<br>0.012-1.694	 .074<br>.123	1.00<br>.223<br>.276	 .019-2.622<br>.022-3.530	 .233<br>.322
N. of metastatic sites 1<br>2<br>>2	3/21<br>1/20<br>2/22	1.00<br>.35<br>1.03	 0.037-3.418<br>0.166-6.410	 .370<br>.974			
Visceral mets no<br>yes	4/35<br>2/28	1.00<br>.866	0.157-4.769	.868			
Locoregional LN mets no<br>yes	2/32<br>4/31	1.00<br>2.75	.501-15.148	.244			
Bone mets metachronous<br>synchronous	6/54<br>0/10	1.00<br>.032	0.000->100	.405			
Surgery for bone mets no<br>yes	5/61<br>1/2	1.00<br>5.98	.666-53.7	.110			
RT for bone mets no<br>yes	0/19<br>6/44	1.00<br>>29	.004->100	.451			
CT for bone mets no<br>yes	1/13<br>5/50	1.00<br>1.253	.146-10.737	.837			
Bone-directed therapies no<br>yes	3/41<br>3/22	1.00<br>1.325	.265-6.621	.732			

M+: metastatic; LA: locally advanced; Lim: limited; Mets: metastases; SRE: skeletal-related event; RT: radiotherapy; CT: chemotherapy; NPC: nasopharyngeal carcinoma; HR: hazard ratio; NA: not assessable.
