# Supplementary material for: Bone metastases from head and neck malignancies: Prognostic factors and skeletal-related events
Source: PLoS One. 2019 Mar 20;14(3):e0213934. doi: 10.1371/journal.pone.0213934 (PMC6426213; doi:10.1371/journal.pone.0213934)
Supplement: S2 Table — M+: metastatic; LA: locally advanced; Lim: limited; Mets: metastases; SRE: skeletal-related event; RT: radiotherapy; CT: chemotherapy; NPC: nasopharyngeal carcinoma; HR: hazard ratio. (PDF) [file pone.0213934.s004.pdf]

Supplementary Table 2. Uni-/Multivariate analyses of predictive factors for SREs in non-NPC patients.

**Variable**	**Univariate analysis**				**Multivariate analysis**		
	SRE/n	HR	95% CI	p	HR	95% CI	p
Age <50<br>≥50	5/17<br>29/108	1.00<br>.794	0.307.2.055	.634			
Sex Male<br>Female	25/99<br>9/26	1.00<br>1.903	0.884-4.098	.10	1.00<br>2.099	.924-4.470	.077
Stage at diagnosis Lim<br>LA<br>M+	6/17<br>19/88<br>9/20	1.00<br>0.490<br>1.371	0.195-1.230<br>0.487-3.856	.129<br>.550			
Histology squamous<br>other	29/107<br>5/21	1.00<br>0.991	0.383-2.568	.986			
N. of metastatic sites 1<br>2<br>>2	6/31<br>10/46<br>18/48	1.00<br>1.09<br>2.24	0.398-3.336<br>0.890-5.661	.087<br>.856	1.00<br>2.538<br>.827	.942-6.840<br>.891-2.351	.721<br>.066
Visceral mets no<br>yes	11/46<br>23/79	1.00<br>1.20	0.587-2.481	.610			
Locoregional LN mets no<br>yes	9/48<br>25/77	1.00<br>2.13	0.992-4.603	.052	1.00<br>1.861	.820-4.223	.137
Bone mets metachronous<br>synchronous	31/118<br>3/10	1.00<br>1.17	0.359-3.853	.788			
Surgery for bone mets no<br>yes	30/120<br>4/5	1.00<br>2.352	.818-6.762	.113			
RT for bone mets no<br>yes	16/57<br>18/68	1.00<br>.824	.418-1.624	.576			
CT for bone mets no<br>yes	13/36<br>21/89	1.00<br>.462	.229-.932	.031	1.00<br>.369	.176-.775	.008
Bone-directed therapies no<br>yes	12/83<br>22/42	1.00<br>3.981	1.968-8.052	< .0005	1.00<br>4.614	2.194-9.286	< .0005

M+: metastatic; LA: locally advanced; Lim: limited; Mets: metastases; SRE: skeletal-related event; RT: radiotherapy; CT: chemotherapy; NPC: nasopharyngeal carcinoma; HR: hazard ratio.
